# Tricuspid Annuloplasty Using a Handmade Gore-Tex Band: A Retrospective Study, Case Series, and Literature Review

**DOI:** 10.7759/cureus.80474

**Published:** 2025-03-12

**Authors:** Selman Dumani, Laureta Dibra, Ermal Likaj, Alessia Mehmeti, Alfred Ibrahimi, Edlira Rruci, Stavri Llazo, Aferdita Veseli, Vera Beca, Ali Refatllari, Altin Veshti

**Affiliations:** 1 Division of Cardiac Surgery, University Hospital Center "Mother Teresa", Tirana, ALB; 2 Division of Anesthesiology, University Hospital Center "Mother Teresa", Tirana, ALB; 3 Division of Nosocomial Infections, University Hospital Obstetrics and Gynecology "Queen Geraldina", Tirana, ALB

**Keywords:** de vega annuloplasty, functional tricuspid valve regurgitation, gore-tex band annuloplasty, tricuspid ring, tricuspid valve annuloplasty, tricuspid valve repair

## Abstract

Background

Tricuspid valve regurgitation is frequently overlooked by cardiologists and cardiac surgeons alike; consequently, the tricuspid valve is often referred to as “the forgotten” valve. It is the most common complication of left heart valve disease. Ring annuloplasty and suture (De Vega) annuloplasty represent two common surgical treatment techniques. We developed a technique to stabilize the tricuspid annulus using a simple handmade Gore-Tex vascular prosthesis. Our literature review did not reveal any publications describing an identical approach.

Objectives

In this article, we report the results of our technique for repairing the tricuspid valve, particularly emphasizing both the short- and long-term echocardiographic outcomes, along with a review of current tricuspid valve repair surgical techniques.

Methods

This retrospective study investigated the outcomes of 10 patients who underwent tricuspid valve repair via the tailoring of a simple Gore-Tex vascular graft and implanting it in the tricuspid annulus. Transthoracic echocardiography was used for the preoperative and postoperative evaluation of tricuspid regurgitation (TR). The minimum postoperative follow-up time was three months, compared with a maximum follow-up period of five years. Echocardiographic follow-up was employed to assess the patency of the tricuspid valve repairs. Data are presented as percentages, means, and standard deviations. Statistical analyses were conducted using SPSS Statistics for Windows, version 26.0 (IBM Corp., Armonk, NY, USA). The primary outcome measures were in-hospital and long-term tricuspid valve competence. We reviewed the literature on tricuspid valve repair to provide a brief overview of this topic.

Results

The study group comprised 10 patients (8 women and 2 men) with a mean age of 62.3 ± 14.46 years. At hospital admission, all patients were categorized as either NYHA (New York Heart Association) functional class III (80%) or IV (20%). Preoperative TR was severe in 70% of patients and moderate in 30%. The mean pulmonary artery pressure was 57.6 ± 14.98 mmHg. In most patients, the primary indication for surgery was mitral valve pathology in 70% of patients, followed by interatrial septal defect in 30% of patients. Immediately after surgery, nine patients exhibited 1+ TR and one patient had 2+ TR. After long-term follow-up, 77.7% of patients had 1+ TR and 22.3% had 2+ TR.

Conclusions

Tricuspid valve repair using handmade Gore-Tex band annuloplasty can be an effective method, yielding excellent early and long-term outcomes regarding repair patency, as assessed by echocardiography. This technique is simple, reproducible, and cost-effective.

## Introduction

Tricuspid regurgitation (TR), characterized by the backflow of blood from the right ventricle into the right atrium during systole, often necessitates surgical intervention when it reaches moderate-to-severe levels [1]. TR can be primary or secondary (functional) to left-sided heart disease or pulmonary hypertension. It is estimated that up to 90% of moderate-to-severe TR in Western countries is due to these causes [2]. Two widely adopted surgical techniques for tricuspid valve repair are the De Vega (suture-based) annuloplasty and ring-based annuloplasty using prosthetic materials. The De Vega procedure involves plicating the tricuspid annulus with sutures to reduce its size, while ring annuloplasty employs a prosthetic ring to provide structural support to the annulus. The primary goal of surgical repair is to restore valve competence and prevent the progression of right heart failure. We report our experience with tricuspid repair using a handmade Gore-Tex band, which ultimately reduces and stabilizes the tricuspid annulus. The primary aim of this study was to investigate both the short-term and long-term echocardiographic outcomes of this surgical technique, supplemented by a brief overview of current tricuspid repair methods as reported in the literature.

## Materials and methods

**Figure 1:**
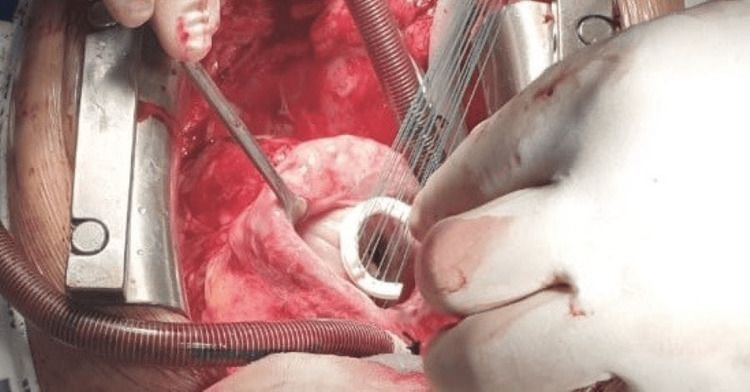
Gore-Tex vascular prosthesis band implanted over tricuspid annulus with Ticron 2/0 sutures

This retrospective study included 10 patients who underwent tricuspid valve (TV) repair for functional (secondary) TR during elective cardiac operations performed between May 2018 and October 2024. Preoperative data, major intraoperative and immediate postoperative adverse events, and early and long-term postoperative echocardiographic findings were assessed. Perioperative data were reviewed from hospital medical files. The surgical technique involved tailoring a simple Gore-Tex vascular graft and implanting it into the tricuspid annulus to mimic ring annuloplasty (Figure 1).

Echocardiography was performed to assess TR severity during the perioperative period and follow-up. A vena contracta width of 3-6.9 mm was defined as moderate TR, whereas a vena contracta width of >7 mm was defined as severe TR [3]. Data are presented as percentages, means, and standard deviations. Statistical analysis was performed using SPSS Statistics for Windows, version 26.0 (IBM Corp., Armonk, NY, USA). We did not create comparative groups because our sample group is small. The primary outcome measures were in-hospital and long-term TV competence following repair. We reviewed several reports on TV repair to provide a brief overview of this issue in the current literature.

## Results

The study cohort included 10(100%) patients, comprising eight women (80%) and two men (20%). The mean age was 62.3 ± 14.46 years. At the time of hospital admission, all patients were classified as NYHA (New York Heart Association) functional class III, with eight (80%) patients, or class IV, with two (20%) patients. Preoperative echocardiographic findings revealed severe and moderate TR in 70% (seven patients) and 30% (three patients) of patients, respectively. The mean pulmonary artery systolic pressure was 57.6 ± 14.98 mmHg. The mean left ventricular ejection fraction was 59.6 ± 8.83%. Right chamber dilation (both right atrium and ventricle) was observed in all patients. Mitral valve pathology was present in 70% (7/10) of patients. Four (40%) patients presented with mitral regurgitation (including one with a patent foramen ovale), whereas mitral stenosis was the primary diagnosis in three (30%) patients. One (10%) patient underwent mitral valve surgery for the second time. Three (30%) patients had interatrial defects (ostium secundum atrial septal defects), with one also exhibiting anomalous pulmonary vein drainage (subtotal abnormal pulmonary venous drainage). The mean extracorporeal circulation time was 116 ± 94.4 minutes, and the mean clamping time was 115 ± 66.6 minutes. The mean hospital stay was 9.9 ± 8.2 days.

During follow-up, two deaths occurred: one on the fourth postoperative day and the other three months after surgery. The first patient, who had undergone mitral valve replacement for the second time, presented with low cardiac output preoperatively and severe pulmonary hypertension. The second patient died due to a cerebral hemorrhage; the final echocardiographic results revealed no TR. Preoperative, intraoperative, and postoperative early outcomes are summarized in Table 1.

**Table 1:** Preoperative ,intraoperative and postoperative early outcomes SD, standard deviation

Data	Mean ± SD or N (%)
Male	2 (20%)
Female	8 (80%)
Age (years)	62.3 ± 14.46
New York Heart Association class III	8 (80%)
New York Heart Association class IV	2 (20 %)
Ejection fraction (%)	59.6% ± 8.83
Pulmonary artery systolic pressure (mmHg)	57.6 ± 14.98
Severe tricuspid regurgitation	6 (60 %)
Moderate tricuspid regurgitation	4 (40%)
Right chamber dilatation	10 (100%)
Prior cardiac surgery	1 (10%)
Simultaneous atrial septal defect	3 (30%)
Simultaneous mitral regurgitation	4 (40%)
Simultaneous mitral stenosis	3 (30%)
Mean extracorporeal circulation time (min)	116 ± 94.4
Mean clamping time (min)	115 ± 66.6
Mean hospital stay (days)	9.9 ± 8.2
Hospital mortality	1 (10 %)

Immediate postoperative assessment showed minimal (1+) TR in eight (80%) patients and mild (2+) in two (20%) patients. The mean pulmonary artery systolic pressure during the postoperative period was 32.5 ± 2.7 mmHg. Recent echocardiograms of the eight surviving patients continue to demonstrate minimal to mild TR (1+ to 2+). Over long-term follow-up, 77.7% of patients exhibited 1+ TR, while 22.3% demonstrated 2+ TR. The preoperative and postoperative echocardiographic data are presented in Table 2.

**Table 2:** Preoperative and early and late postoperative echocardiographic

Tricuspid regurgitation	Preoperative, N (%)	Immediate postoperative, N (%)	Follow-up, N (%)
Grade 1+	0 (0 %)	9 (90%)	7 (77.7 %)
Grade 2+	0 (0%)	1 (10%)	2 (22.3 %)
Grade 3+	3 (30%)	0 (0 %)	0 (0 % )
Grade 4+	7 (70%)	0 (0%)	0 (0 % )

## Discussion

The classical surgical techniques for repairing tricuspid valve - De Vega suture annuloplasty and ring annuloplasty - yield excellent outcomes with regard to early postoperative regurgitation. However, long-term durability and freedom from reintervention remain key points of comparison between these approaches. In our clinic, we have been employing De Vega suture annuloplasty for decades and have recently adopted a handmade Gore-Tex band technique to stabilize the tricuspid annulus. This discussion focuses on the efficacy, durability, procedural complexity, and cost considerations of both techniques while exploring the factors that contribute to the favorable outcomes achieved with our method.

In a retrospective study by Guenther et al. involving 717 consecutive patients who underwent TV surgery between 1975 and 2009 with either a ring annuloplasty (n = 433) or a De Vega suture annuloplasty (n = 255) [4], freedom from TV reoperation at 10 years after repair was reported as 87.9% ± 3% for De Vega annuloplasty compared with 98.4% ± 1% for ring annuloplasty. Furthermore, the 10-year survival rates after TV repair were 39% ± 3% and 46% ± 7% for the De Vega and ring annuloplasty groups, respectively, while the 30-day mortality rates were 15.7% for the De Vega group and 13.8% for the ring annuloplasty group. Tang et al. further determined annuloplasty ring placement to be an independent predictor of both long-term survival and event-free survival. The ring group demonstrated significantly better long-term survival, event-free survival, and freedom from recurrent TR, with a tendency toward fewer TV reoperations. Multivariable analysis revealed that the use of an annuloplasty ring independently predicted both long-term survival (hazard ratio 0.7; 95% confidence interval 0.5 to 1.0; P = 0.03) and event-free survival (hazard ratio 0.8; 95% confidence interval 0.6 to 1.0; P = 0.04) [5].

While some investigators advocate for ring annuloplasty, emphasizing its potential to enhance survival and long-term durability, some research has found no significant differences between the two techniques. In a recent study, Omran and Zahra compared De Vega annuloplasty with ring annuloplasty for functional TR repair performed during mitral valve surgery (N = 66). The extent of TR immediately postoperatively (P = 0.163) and at one postoperative year (P = 0.119) were comparable between the groups [6]. Other studies have concluded that the differences between the two procedures are minimal or not statistically significant [7,8], although a few reports support the superiority of the De Vega technique.

In a retrospective study that did not directly compare the two techniques, the De Vega procedure was deemed safe and reliable, reporting a 15-year survival rate of 74.0%, a 15-year freedom from reoperation rate of 91.6%, and a 15-year freedom from all events rate of 58.7% [9]. Furthermore, a retrospective comparative study of 299 patients with rheumatic TR treated surgically with prosthetic ring annuloplasty, commissurotomy, and suture annuloplasty revealed that, on univariate analysis, late mortality was significantly higher in association with tricuspid flexible Duran ring annuloplasty than with suture annuloplasty (43% vs. 26.4%, P < 0.05). That study also identified the use of a tricuspid Duran flexible ring annuloplasty as a significant risk factor for reoperation, with rates of 44.3% compared to 21.2% for tricuspid suture annuloplasty (P < 0.005) [10].

In a large meta-analysis, Parolari et al. found that freedom from moderate TR was significantly greater in patients undergoing ring annuloplasty (78.9% ± 5.0% at 15 years vs. 60.0% ± 4.2%, log-rank P = 0.0107). Ring annuloplasty was associated with superior outcomes, serving as a protective factor against early death and long-term recurrence of TR. This meta-analysis further demonstrated that prosthetic ring annuloplasty yields better outcomes compared with suture-based techniques, particularly in terms of long-term recurrence of TR. Moreover, available data suggest that rigid rings provide enhanced protection against recurrent TR at follow-up and contribute to improved early survival [11].

Another noteworthy contribution to the evidence on TV repair, comparing ring annuloplasty with De Vega suture annuloplasty, was the review by Khorsandi et al., which selected 14 studies from a total of 306 reviewed according to a structured protocol [12]. The findings strongly support the use of ring annuloplasty over the De Vega technique for moderate-to-severe TR, particularly in terms of lower TR recurrence rates necessitating reoperation and improved long-term survival. Furthermore, the absence of ring application emerged as a predictor of tricuspid repair failure leading to reoperation, underscoring the critical role that stabilization of the TV annulus plays as a preventive measure against long-term repair failure.

With regard to the technique employed in our study, we found no previous reports that have applied the same method. One publication that appears to be closely related is the study by Chang et al. In their retrospective cohort study involving 334 patients who underwent tricuspid annuloplasty for functional TR between 1997 and 2006, 117 patients underwent De Vega suture annuloplasty, while 217 underwent autologous pericardial strip annuloplasty. The mean follow-up period was 3.5 years, and at discharge, 34 (11.1%) patients continued to exhibit significant TR. The study observed that TR grade appeared to be getting worse in the conventional De Vega suture annuloplasty group in comparison with the group of patients who underwent pericardial strip annuloplasty (P = 0.05). Moreover, recurrence-free survival was significantly higher in the pericardial strip group than in the De Vega group, with rates of 86.8% and 71.9%, respectively (P = 0.039) [13]. Our technique similarly aims to reinforce the tricuspid annulus.

In summary, the available evidence suggests that ring annuloplasty is favored over the De Vega technique, as indicated by both superior echocardiographic outcomes and improved long-term survival. Technically, the stabilization provided by the ring appears to be a crucial factor, and our results reflect that our method effectively achieves this goal. An additional advantage of our technique is its simplicity, reproducibility, and cost-effectiveness, rendering it a viable surgical option. Nonetheless, our study was limited by its small sample size, and larger studies are warranted to confirm these promising findings.

Limitations of our study

The main limitation is related to the small sample group. The second limitation is the fact that because our group was small, we did not create a comparison group.

## Conclusions

Tricuspid valve regurgitation is often related to mitral valve pathologies and atrial septal defects. The most common repair surgical techniques used to repair TR are De Vega suture and ring annuloplasty. Current evidence suggests that ring annuloplasty may offer superior durability and a lower rate of TR recurrence relative to the De Vega technique, although the increased complexity and cost associated with ring annuloplasty must be weighed against its potential benefits. Tricuspid valve repair using our technique, handmade Gore-Tex band annuloplasty technique, represents a promising approach. It imitates the ring annuloplasty, offering a stabilization of the tricuspid annulus that is reported to be a crucial role in the long-term results of repair. Our results demonstrated excellent early and long-term outcomes in terms of repair patency, as evaluated by echocardiography. Our technique, which emulates ring annuloplasty, is safe, reproducible, and cost-effective. While larger studies are needed to provide more robust data, in the absence of conventional tricuspid rings, this method may constitute a viable option.
